# Casticin inhibits self-renewal of liver cancer stem cells from the MHCC97 cell line

**DOI:** 10.3892/ol.2014.1972

**Published:** 2014-03-14

**Authors:** GUICHENG HE, XIAOCHENG CAO, MENG HE, XIFENG SHENG, YOUHUA WU, XIAOHONG AI

**Affiliations:** 1 Department of Oncology, First Affiliated Hospital of University of South China, Hengyang, Hunan 421001, P.R. China; 2 Medical College, Hunan Normal University, Changsha, Hunan 410013, P.R. China

**Keywords:** hepatocellular carcinoma, cancer stem cells, casticin, self-renewal, β-catenin

## Abstract

Casticin exerts anticarcinogenic activity in several types of cancers, including human hepatocellular carcinoma (HCC). The aim of the present study was to investigate the effects of casticin, which is derived from *Fructus Viticis Simplicifoliae*, on the self-renewal capacity of liver cancer stem cells (LCSCs) derived from the HCC MHCC97 cell line. The present study demonstrated that casticin significantly inhibited the proliferation of LCSCs from the MHCC97 cell line in a dose-dependent manner (P<0.05), the half maximal inhibitory concentration of the parental cells and LCSCs was 17.9 and 0.5 μmol/l, respectively. Furthermore, casticin reduced the sphere-forming capacity of LCSCs and downregulated β-catenin protein expression in a concentration-dependent manner. Lithium chloride, an agonist known to activate the Wnt/β-catenin signaling pathway, attenuated the casticin-induced downregulation of β-catenin protein expression and inhibited the self-renewal capacity. To the best of our knowledge, the present study is the first to demonstrate that casticin effectively eradicates LCSCs and β-catenin was identified as the potential target. Thus, casticin may offer a novel therapeutic approach for the treatment of HCC.

## Introduction

Hepatocellular carcinoma (HCC) is a common malignancy with ~600,000 new cases of primary liver cancer each year and is the third leading cause of cancer-related mortality worldwide (1,2). The prognosis of HCC depends on the stage of cancer at diagnosis. Although surgery has resulted in an improved five-year survival rate of select patients, the majority of patients with HCC gain no significant benefit from traditional chemotherapy (3). HCC is mostly resistant to conventional chemotherapy and radiotherapy, and commonly metastasizes to lymph nodes, lungs, bones and adrenal glands, as well as the skull (4). Therefore, identifying novel therapeutic strategies for the treatment of HCC is crucial.

Cancer stem cells (CSCs) represent a small subset of tumor cells with stem cell properties, and are able to initiate and sustain tumor growth (5,6). Similar to somatic stem cells, CSCs possess the characteristics of self-renewal, differentiation and proliferation following a prolonged period of quiescence (7). Furthermore, CSCs are responsible for the failure of chemotherapy and radiotherapy, as well as the initiation, progression and recurrence of local and distant metastasis. Therefore, the clinical corollary currently extends to proposals of cancer treatment via targeting putative CSCs.

Previous studies have focused on identifying the characteristics of CSCs in HCC, specifically in liver cancer stem cells (LCSCs). Markers that characterize putative human LCSCs, such as cluster of differentiation (CD)133, CD90, CD44, epithelial cell adhesion molecule, OV6 and CD13 have previously been investigated (8–13). Cell marker expression is associated with fetal liver cell marker expression, tumor initiation, *in vitro* culture and chemoresistance. Cells lacking such markers exhibit LCSC properties; therefore, individual markers may not be sufficient to represent all of the characteristics of CSCs (14,15).

Novel therapeutic agents are urgently required for the treatment of HCC. Casticin (3′,5-dihydroxy-3,4′,6,7-tetramethoxyflavone), also known as vitexicarpin, is the predominant component of *Fructus Viticis*, a traditional Chinese medicine prepared from the fruit of *Vitex trifolia L.*, which has been widely used in China, for thousands of years, as an anti-inflammatory agent and for the treatment of certain cancers (16). Casticin inhibits prolactin release *in vivo* and *in vitro* (17), induces leukemic cell death via apoptosis and mitotic catastrophe, and synergizes with phosphatidylinositol 3-kinase (18). Recent studies have demonstrated the anticarcinogenic properties of casticin; Yang *et al* (19) reported that casticin significantly induced apoptosis of HCC cells and may affect the number of glioma stem-like cells that were sorted from U251 cells (20). However, the function of casticin in regulating the self-renewal capacity of LCSCs has not been fully investigated.

The present study aimed to demonstrate that casticin results in significant inhibition of the self-renewal capacity of CD133^+^ sphere-forming cells (SFCs) of the MHCC97 cell line, namely LCSCs, by downregulating β-catenin expression.

## Materials and methods

### Cell culture and reagents

The HCC MHCC97 cell line was purchased from Shanghai Xiangf Biotechnology Co., Ltd. (Shanghai, China). The MHCC97 cells were maintained in Dulbecco’s modified Eagle’s medium (DMEM) supplemented with 10% fetal bovine serum (FBS; Hangzhou Sijiqing Biological Engineering Materials Co., Ltd, Hangzhou, China), 100 U/ml penicillin and 100 μg/ml streptomycin (Invitrogen Life Technologies, Carlsbad, CA, USA), and incubated in an atmosphere of 5% CO_2_ at 37°C. Casticin was purchased from Chengdu Biopurify Phytochemicals Ltd. (Chengdu, China, dissolved in dimethyl sulfoxide (DMSO) as a 10-mmol/l stock solution, and diluted in a medium to the indicated concentration. MTT and lithium chloride were purchased from Sigma-Aldrich (St. Louis, MO, USA). Trypsin and DMSO were purchased from Amersco Company (Solon, OH, USA). Mouse anti-human β-catenin, cyclin D1, β-actin antibodies and horseradish peroxidase-conjugated rabbit anti-mouse secondary antibody were all purchased from Santa Cruz Biotechnology, Inc. (Santa Cruz, CA, USA).

### Cell sorting and sphere culture

Cell sorting was performed with MHCC97 cells using the cell surface phenotype, CD133^+^, through magnetic activated cell sorting (MACS) separation columns (Miltenyi Biotec, Bergisch Gladbach, Germany) according to the manufacturer’s instructions. Cells were trypsinized and washed with phosphate-buffered saline (PBS) and suspended in PBS containing 0.5% bull serum albumin (BSA). FcR Blocking Reagent (100 μl; anti-CD133 antibody), 100 μl CD133-conjugated MicroBeads (AC133 Cell Isolation Kit, Miltenyi Biotec), and 10^8^ cells were subsequently added to the sample and incubated in parallel for 30 min on ice. After washing the cells, CD133-positive and -negative fractions were isolated through MACS separation columns. The CD133^+^ and parental cells were collected and washed to remove serum, and suspended in serum-free DMEM/F12, which was supplemented with 20 ng/ml human recombinant epidermal growth factor (EGFR), 20 ng/ml human recombinant basic fibroblast growth factor, 2% B27 supplement without vitamin A, 0.4% BSA, 4 ng/ml insulin, 100 IU/ml penicillin and 100 μg/ml streptomycin. The single-cell suspensions were suspended at a density of 2,000 cells/ml in stem cell-conditioned medium and seeded into ultra-low attachment six-well plates (Corning, Inc., Corning, NY, USA). When the spheroid diameter reached 50 μm, the suspension cultures were passaged every six days. Colonies were counted in 10 different views under a microscope (IX71, Olympus, Tokyo, Japan). The volume of the spheroids (μm^3^) was estimated using the following formula: V=(4/3) πR^3^, where R denotes radius. The experiments were repeated three times in duplicate.

### Flow cytometry (FCM)

The parental cells, and sorted CD133^+^ and CD133^−^ cells were resuspended in PBS, sub-packaged in Eppendorf tubes (density, 1×10^5^ cells/ml) and incubated directly with the conjugated monoclonal antibodies, mouse anti-human CD133-R-phycoerythrin (PE) and mouse IgG2b isotype control-PE for 30 min at 4°C in the dark. The fluorescence value was measured by FCM with 10,000 cells per tube.

### Spheroid passage and sphere formation assay

The CD133^+^ SFCs of the MHCC97 cell line were collected by gentle centrifugation at 80 × g (TL-5-A, Jintan Shenglan Instrument Manufacturing Co., Ltd., Jintan, China), dissociated with trypsin-EDTA and mechanically disrupted using a pipette. The resulting single cells were centrifuged to remove the enzyme, resuspended in a stem cell-conditioned culture medium and allowed to reform spheres. The tumorspheres were passaged every six days until reaching a diameter of 50 μm. The dissociated single SFCs were diluted to a density of 500 cells/ml, the diluted cell suspension was plated onto an ultra-low attachment 96-well plate (Corning Inc.) with 2 μl/well of serum-free medium (150 μl). The wells containing only one cell were marked, observed and photographed with an inverted microscope (IX71, Olympus) daily for approximately nine days.

To examine the effects of casticin on sphere formation, the resulting single-cell suspension, with a density of 2×10^3^ cells/ml, was plated onto an ultra-low attachment six-well plate supplemented with serum-free medium at the same volume as the primary tumorsphere formation experiment (primary experiment). As a second experimental process the density was altered to ~1×10^3^ cells/ml. In the primary experiment the medium was supplemented with various concentrations of casticin, however, this was not the case in the second experiment.

In order to assess the effect of the Wnt/β-catenin pathway on the formation of sub-tumorspheres, dissociated MHCC97 CD133^+^ SFCs were treated with a culture medium containing casticin-LiCl (0 μmol/l, 20 mmol/l), casticin-LiCl (1 μmol/l, 0 mmol/l), casticin-LiCl (1 μmol/l, 10 mmol/l), casticin-LiCl (1 μmol/l, 20 mmol/l) or control, 0.1% DMSO respectively, for 24 h and the formation of sub-tumorspheres was observed.

### In vivo tumorigenicity assay

Twenty pathogen-free male Balb/c-nu mice (age, 5–6 weeks) were purchased from the Animal Institute of the Chinese Academy of Medical Science. The animal studies were performed in accordance with standard protocols approved by the Ethics Committee of Hunan Normal University and the Committee of Experimental Animal Feeding and Management (Changsha, China). The mice were randomly divided into five groups (n=4 per group) and maintained under standard conditions, according to typical protocols. The cells were suspended in a serum free-DMEM/Matrigel (BD Biosciences, Franklin Lakes, NJ, USA) mixture (1:1 volume). The mice were inoculated with different quantities of CD133^+^ SFCs (5×10^2^, 1×10^3^, 5×10^3^, 1×10^4^ and 5×10^4^ cells) in one flank, and unsorted MHCC97 cells (5×10^4^, 1×10^5^, 2×10^5^, 5×10^5^ and 1×10^6^ cells) in the other. Tumorigenicity experiments were terminated two months after cell inoculation. Tumor size was measured using a caliper and the volume was calculated as follows: V (mm^3^) =L × W^2^ × 0.5, where L denotes length and W denotes width. The harvested tumors were photographed and weighed immediately. Specimens from tumor tissue samples were fixed in 10% neutral-buffered formalin, processed in paraffin blocks and sectioned. The sections were stained with hematoxylin and eosin (H&E) and examined under an inverted microscope (IX71, Olympus).

### MTT assay

CD133^+^ SFCs or parental MHCC97 cells were seeded in 96-well plates pre-coated with 0.6% agarose at a density of 5,000 cells/well as described previously (19). One day after plating, 8-bromo-7-methoxychrysin of different concentrations was added to each well and cultured for 48 h at 37°C. Following removal from the medium, the cells were incubated with 5 mg/ml MTT for 4 h. The cells were extracted with acidic isopropanol and the absorbance at a 570-nm wavelength (A570) was measured using an enzyme-labeling instrument (ELx800 Absorbance Microplate Reader type, BioTek Instruments, Inc., Winooski, VT, USA). The relative cell proliferation inhibition rate was calculated as follows: Average A570 of the experimental group/average A570 of the control group × 100%.

### Western blot analysis

The preparation of whole cell lysates and western blot analysis were performed as previously described (19). Mouse anti-human β-catenin, cyclin D1 and β-actin antibodies served as primary antibodies. The signals were visualized using a chemiluminescent substrate (enhanced chemiluminescence; Amersham Life Science, Arlington Heights, IL, USA) and β-actin served as an internal control. Images were scanned and densitometry analysis was performed with a UN-SCAN-IT graph digitizer (Silk Scientific, Inc., Orem, UT, USA).

In order to assess the effect of LiCl attenuated during the casticin-induced downregulation of β-catenin or cyclin D1 protein expression, dissociated MHCC97 CD133^+^ SFCs were treated with a culture medium containing casticin-LiCl (0 μmol/l, 20 mmol/l), casticin-LiCl (1 μmol/l, 0 mmol/l), casticin-LiCl (1 μmol/l, 10 mmol/l), casticin-LiCl (1 μmol/l, 20 mmol/l) or control, 0.1% DMSO, respectively, for 24 h and the expression of β-catenin or cyclin D1 was observed.

### Statistical analysis

The data are expressed as means ± SD and the data were analyzed with SPSS software, version 15.0 (SPSS, Inc., Chicago, IL, USA). In addition, one-way analysis of variance was performed. After the equal check of variance, two-two comparisons of the means between the test and control groups were performed using the least-significant difference method; or Dunnett’s test was used. P<0.05 was considered to indicate a statistically significant difference.

## Results

### Isolation and identification of LCSCs from the MHCC97 cell line

**Figure 1:**
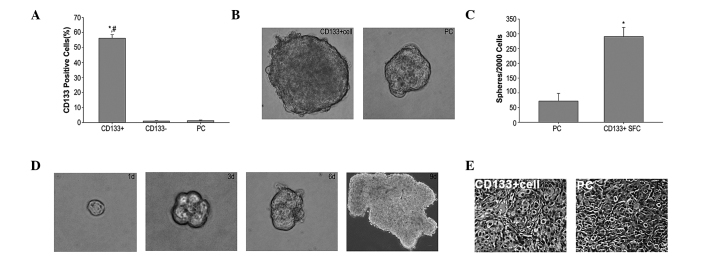
CD133^+^ SFCs derived from the MHCC97 cell line possess characteristics of LCSCs. (A) CD133^+^ cell subpopulation, sorted from the hepatocellular carcinoma MHCC97 cell line by magnetic activated cell sorting, overexpressed the stem cell surface marker, CD133, detected by flow cytometry using PE-conjugated anti-human CD133 antibody. ^#^P<0.05 compared with the CD133^−^ cell group. ^*^P<0.05 compared with the PCs(B and C) CD133^+^ cells derived from the MHCC97 cells and PCs formed liver cancer spheroids in stem cell-conditioned medium (magnification, ×100). Data are expressed as the mean ± standard deviation (n=3). ^*^P<0.05 compared with the PCs. (D) The sphere formation of single cells in six-well plates was detected on the first, third, sixth (magnification, ×400) and ninth day (magnification, ×40). (E) Hematoxylin and eosin staining revealed histological characteristics in tumor xenografts derived from CD133^+^ SFCs comparable with the PCs (magnification, ×100). CD133, cluster of differentiation 133; SFCs, sphere-forming cells; LCSCs, liver cancer stem cells; PE, R-phycoerythrin; PC, parental cell; d, days.

CD133 is classified as a CSC marker in HCC (10). Therefore, the CD133^+^ subpopulation was sorted from the MHCC97 cell line using MACS and cultured *in vitro*. The expression of the stem cell marker, CD133, was examined by FCM. The subpopulation of CD133^+^ cells showed a high purity of 56.26±2.34% compared with the purity of CD133^−^ (1.04±0.27%) and parental cells (3.32±0.38%; Fig. 1A). To establish long-term cultures enriched in stem cells from sorted CD133^+^ cells, the tumorsphere formation assay in a stem cell-conditioned medium was performed. The spheroids from CD133^+^ and parental cells were obtained after six days of culture (Fig. 1B). The CD133^+^ subpopulation exhibited a greater quantity of tumorsphere formation and increased size compared with the parental cells (Fig. 1B and C). These findings indicate the existence of LCSCs in sorted CD133^+^ cells and that LCSCs are highly enriched in CD133^+^ tumor-forming cells.

To further investigate stem-cell properties and the function of CD133^+^ SFCs, self-renewal capacity and tumorigenic potential were analyzed. The capacity of single cells (obtained from CD133^+^ dissociated spheres) to form secondary tumorspheres was measured. Within nine days of culture, new spheroids of growing undifferentiated CD133^+^ cells were observed (Fig. 1D). Thus, the *in vitro* CD133^+^ SFCs from the MHCC97 cell line demonstrated a self-renewing capacity. Furthermore, the tumorigenic potential of CD133^+^ SFCs of the MHCC97 cell line was investigated in Balb/c-nu mice. Our findings demonstrated that ≤2×10^5^ parental cells were required to initiate stable tumor formation 37 days after inoculation. By contrast, only 1×10^3^ CD133^+^ SFCs were sufficient to generate visible tumors 27 days after inoculation (Table I). These data indicate that CD133^+^ SFCs of the MHCC97 cell line have a greater tumerogenic capacity compared with parental cells *in vivo*. Additionally, H&E staining revealed histological characteristics in tumor xenografts, which were derived from CD133^+^ SFCs, were similar to those of the parental cells (Fig. 1E). Collectively, these data demonstrate that CD133^+^ SFCs possess an ability to self-renew *in vitro* and initiate tumor growth *in vivo*, indicating that the CD133^+^ SFCs may provide a true representation of LCSCs in the HCC MHCC97 cell line.

### Casticin inhibits proliferation and self-renewal of LCSCs derived from the MHCC97 cell line

**Figure 2:**
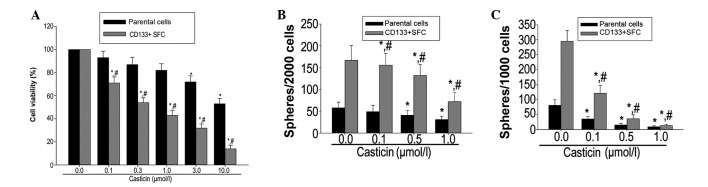
Casticin preferentially inhibits proliferation and self-renewal of LCSCs derived from the MHCC97 cell line. (A) Casticin preferentially inhibited proliferation of CD133^+^ SFCs of the MHCC97 cell line. (B) SFCs were incubated with various concentrations of casticin (0.1, 0.5 and 1.0 Mmol/l) or DMSO for six days. (C) In the absence of casticin, the number of secondary tumorspheres derived from primary tumorspheres decreased compared with the control. Data are expressed as the mean ± standard deviation (n=3). ^*^P<0.05 compared with treatment with the corresponding 0.1% dimethyl sulfoxide treatment group; ^#^P<0.05 compared with the parental cells treated with corresponding concentration of casticin. LCSCs, liver cancer stem cells; CD133, cluster of differentiation 133; SFCs, sphere-forming cells.

CD133^+^ SFCs and parental cells were treated with different concentrations of casticin (0.1, 0.3, 1.0, 3.0 and 10.0 μmol/l) to examine its effect on cell viability of LCSCs using the MTT assay. Casticin preferentially inhibited cell viability of CD133^+^ SFCs derived from MHCC97 cells in a dose-dependent manner (Fig. 2A). The half maximal inhibitory concentration of the parental cells and the CD133^+^ SFCs was 17.9 and 0.5 μmol/l, respectively (Fig. 2A).

In order to evaluate whether casticin suppresses the self-renewal of LCSCs derived from the MHCC97 cell line *in vitro*, the primary tumorspheres were treated with various concentrations of casticin, followed by drug removal and culturing in another passage to form secondary spheres. Treatment with casticin resulted in a decreased number of tumorspheres in LCSCs (Fig. 2B) and a decreased number of secondary tumorspheres; these findings are consistent with the reduced self-renewal capacity of LCSCs by casticin treatment (Fig. 2C).

### Casticin inhibits self-renewal in LCSCs through modulating β-catenin expression

**Figure 3:**
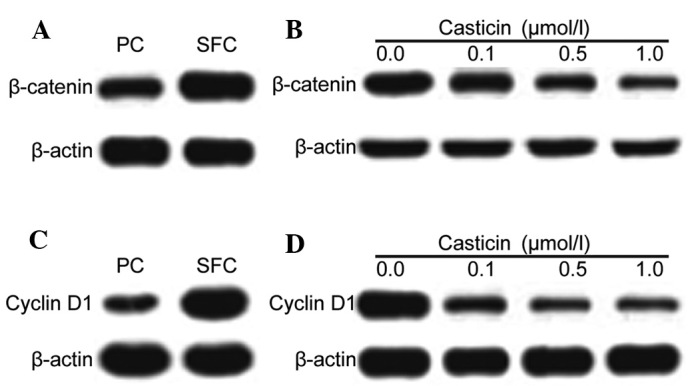
Casticin inhibits β-catenin and cyclin D1 protein expression in LCSCs derived from the MHCC97 cell line. (A) β-catenin is highly expressed in CD133^+^ SFCs compared with corresponding PCs. (B) Casticin downregulated the expression of β-catenin in CD133^+^ SFCs of the MHCC97 cell line. (C) Cyclin D1 is highly expressed in CD133^+^ SFCs compared with corresponding PCs. (D) Casticin downregulated the expression of cyclin D1 in CD133^+^ SFCs of the MHCC97 cell line. LCSCs, liver cancer stem cells; CD133, cluster of differentiation 133; SFCs, sphere-forming cells; PC, parental cell.

The Wnt/β-catenin signaling pathway is a well-established and significant regulator of stem cell self-renewal. Wnt/β-catenin signaling has been implicated in the maintenance of CSCs that are present in liver cancer (21). The expression level of the stem cell signal molecule, β-catenin, and its downstream target molecule, cyclin D1, were measured following casticin treatment in LCSCs and parental cells. Western blot analysis revealed that β-catenin and cyclin D1 were highly expressed in LCSCs compared with the parental cells. Additionally, casticin treatment (0.1, 0.5 and 1.0 μM) resulted in a significant decrease in β-catenin and cyclin D1 expression in LCSCs (Fig. 3).

**Figure 4:**
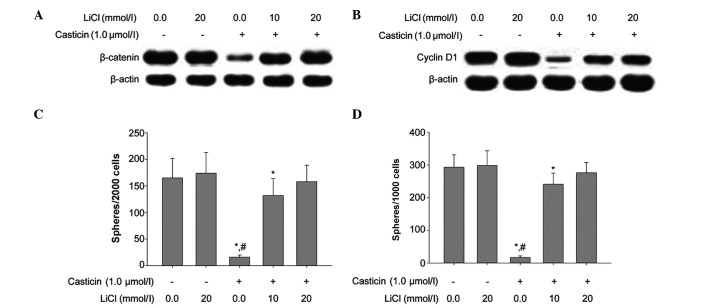
LiCl treatment antagonized the inhibitory effects of casticin on the self-renewal of LCSCs derived from the MHCC97 cell line. Effects of casticin and/or LiCl on expression of (A) β-catenin and (B) cyclin D1. (C and D) Effects of casticin and/or LiCl on the number of tumorspheres formed from CD133^+^ SFCs of the MHCC97 cell line. ^*^P<0.05 compared with treatment with the corresponding 0.1% dimethyl sulfoxide treatment group; ^#^P<0.05 compared with the presence of LiCl cells treated with casticin in the absence of LiCl. LCSCs, liver cancer stem cells; CD133, cluster of differentiation 133; SFCs, sphere-forming cells; LiCL, lithium chloride.

The role of β-catenin in maintaining the self-renewal characteristics of LCSCs was investigated. LCSCs were treated with lithium chloride, an agonist known to activate the Wnt/β-catenin pathway. The addition of lithium chloride resulted in the upregulation of β-catenin and cyclin D1 in LCSCs. In addition, lithium chloride antagonized the inhibitory effects of casticin on the self-renewal of LCSCs and attenuated the casticin-induced downregulation of β-catenin and cyclin D1 expression in LCSCs (Fig. 4).

## Discussion

Selectively targeting CSCs is a focus of investigation with emerging evidence demonstrating their role in the development of cancer (22). A number of potential CSC therapeutic targets have been identified, including the ABC superfamily, anti-apoptotic factors, detoxifying and DNA repair enzymes, and distinct oncogenic cascades (such as the Wnt/β-catenin, hedgehog, EGFR and Notch pathways) (23,24). Certain studies have reported a therapeutic strategy that may successfully kill CSCs; however, certain methods remain under preclinical and clinical evaluation.

Casticin, a promising candidate agent, has been reported to effectively eliminate induced apoptosis and exert antimitotic affects, which results in growth inhibition of cancer cells in different human malignant tumors *in vivo* and *in vitro* (25,26). Feng *et al* (20) proposed that casticin inhibits the proliferation of CSCs. However, the number of studies regarding casticin-targeting CSCs remains limited. Therefore, the effect of casticin on CSCs was investigated in the present study.

Abundant evidence has demonstrated the presence of CSCs in solid tumors. The cell surface marker, CD133, has been used to isolate and identify populations of LCSCs (27). However, it was proposed that individual markers should not be used to represent all of the characteristics of CSCs (15,16). Thus, in the present study, CD133^+^ cells were isolated from the HCC MHCC97 cell line using MACS. The CD133^+^ cells formed anchorage-independent three-dimensional spheres in the stem cell-conditioned culture medium. The self-renewal capacity of CD133^+^ SFCs of the MHCC97 cell line was assessed using a sphere formation assay. The standard criterion for estimating tumorigenicity of tumor cells is with a xenotransplantation assay. The CD133^+^ SFCs of the MHCC97 cell line were assessed for their tumor-initiating ability by subcutaneous inoculation in nude mice. Our findings demonstrated that only 1×10^3^ CD133^+^ SFCs of the MHCC97 cell line were required to initiate tumor growth compared with 5×10^5^ parental cells. These findings identified that the ability of CD133^+^ SFCs to stimulate tumor growth was higher compared with parental cells. However, the two cell subpopulations possessed similar histological characteristics, indicating that CD133^+^ SFCs possess the properties of LCSCs. These findings are consistent with those of Ma *et al* (28).

In the present study, LCSCs were treated with various concentrations of casticin and the influence of casticin on the cell viability and self-renewal capacity were observed. Casticin preferentially inhibited the viability of lower survival percentages compared with the parental cells, a finding that is consistent with that of Feng *et al* (20). Additionally, when the CD133^+^ SFCs were treated with casticin, the sphere-forming capacity was reduced in the primary and secondary generations. Therefore, we hypothesized that casticin preferentially inhibits proliferation and self-renewal of LCSCs.

The classic Wnt/β-catenin signaling pathway is vital in the self-renewal and differentiation of LCSCs, and acts as the predominant factor for chemotherapy resistance. PKF118–310 inhibits the self-renewal of breast tumor-initiating cells by Wnt/β-catenin signaling and CDH11 was found to inhibit actin stress fiber formation, thus, further inhibiting tumor cell migration and invasion via the regulation of Wnt/β-catenin signaling (29). In the present study, the expression of β-catenin and cyclin D1 was higher in the parental cells; when CD133^+^ SFCs of the MHCC97 cell line were treated with casticin, the expression of β-catenin and cyclin D1 was downregulated in a dose-dependent manner. In addition, treatment with lithium chloride effectively attenuated the inhibition of the self-renewal capacity by casticin in CD133^+^ SFCs of the MHCC97 cell line. Our findings indicate that casticin regulates self-renewal of LCSCs by downregulating the expression of β-catenin.

In conclusion, CD133^+^ SFCs of the MHCC97 cell line possess the characteristics of LCSCs. Moreover, casticin inhibited the self-renewal capacity of LCSCs, which was a result of blocking the Wnt/β-catenin signaling pathway. Therefore, casticin, by targeting LCSCs, may have a therapeutic role in the treatment of HCC.

## Figures and Tables

**Table I:** Tumorigenicity experiments of CD133^+^ SFCs and parental cells in Balb/c-nu mice (n=4 per group).

Cell type	Cell number	Tumor incidence	Latency (days)
Parental cells	5×10^4^	0/4	-
	1×10^5^	0/4	-
	2×10^5^	3/4	37
	5×10^5^	4/4	29
	1×10^6^	4/4	7
CD133^+^ SFCs	5×10^2^	0/4	-
	1×10^3^	3/4	27
	5×10^3^	4/4	16
	1×10^4^	4/4	10
	5×10^4^	4/4	7

CD133, cluster of differentiation 133; SFCs, sphere-forming cells.
